# ZnO/PDA/Mesoporous Cellular Foam Functionalized Thin-Film Nanocomposite Membrane towards Enhanced Nanofiltration Performance

**DOI:** 10.3390/membranes13050486

**Published:** 2023-04-29

**Authors:** Jenny Nambikkattu, Anoopa Ann Thomas, Noel Jacob Kaleekkal, Thanigaivelan Arumugham, Shadi W. Hasan, Saravanamuthu Vigneswaran

**Affiliations:** 1 Membrane Separation Group, Department of Chemical Engineering, National Institute of Technology Calicut (NITC), Kozhikode 673601, India; 2 Department of Chemical Engineering, Khalifa University, Abu Dhabi P.O. Box 127788, United Arab Emirates; 3 Center for Membranes and Advanced Water Technology (CMAT), Khalifa University of Science and Technology, Abu Dhabi P.O. Box 127788, United Arab Emirates; 4 Centre for Technology in Water and Wastewater, School of Civil and Environmental Engineering, University of Technology Sydney, Sydney, NSW 2007, Australia; 5 Faculty of Sciences &amp, Technology (RealTek), Norwegian University of Life Sciences, P.O. Box 5003, 1432 As, Norway

**Keywords:** nanofiltration membrane, mesostructured cellular foam, thin-film nanocomposite, 2,4-dichlorophenol, desalination, antifouling

## Abstract

Thin-film nanocomposite (TFN) membranes are the third-generation membranes being explored for nanofiltration applications. Incorporating nanofillers in the dense selective polyamide (PA) layer improves the permeability–selectivity trade-off. The mesoporous cellular foam composite Zn-PDA-MCF-5 was used as a hydrophilic filler in this study to prepare TFN membranes. Incorporating the nanomaterial onto the TFN-2 membrane resulted in a decrease in the water contact angle and suppression of the membrane surface roughness. The pure water permeability of 6.40 LMH bar^−1^ at the optimal loading ratio of 0.25 wt.% obtained was higher than the TFN-0 (4.20 LMH bar^−1^). The optimal TFN-2 demonstrated a high rejection of small-sized organics (>95% rejection for 2,4-dichlorophenol over five cycles) and salts—Na_2_SO_4_ (≈95%) > MgCl_2_ (≈88%) > NaCl (86%) through size sieving and Donnan exclusion mechanisms. Furthermore, the flux recovery ratio for TFN-2 increased from 78.9 to 94.2% when challenged with a model protein foulant (bovine serum albumin), indicating improved anti-fouling abilities. Overall, these findings provided a concrete step forward in fabricating TFN membranes that are highly suitable for wastewater treatment and desalination applications.

## 1. Introduction

The shortage of safe drinking water and the increase in pollution of water bodies are some of the most serious issues confronting the world today [1]. Water resources should be protected to address this concern, and water recovery from wastewater or seawater desalination is viable [2]. Membrane-based separation techniques have become highly relevant in water separation and purification as they are faster and environmentally benign [3]. Nanofiltration (NF)/reverse osmosis (RO) membranes are commonly used for seawater desalination and for the removal of heavy metals and organic contaminants (with a molecular weight cut-off (MWCO) range of 50–1000 Da or 200–1000) [4]. The NF membrane has garnered significant interest in recent years because it produces a higher water flux at low operating pressure and has an excellent separation rate against multivalent salts and small molecules [5].

To date, the state-of-the-art NF membranes are based on a polymeric thin-film composite (TFC) structure that contains a porous base membrane formed via non-solvent induced phase separation (NIPS) and a thin polyamide (PA) selective top layer (<500 nm) created by the interfacial polymerization (IP) of amine and acyl chloride monomers [6]. Even so, the high energy and capital costs of these membranes due to their low permeability and fouling have made industrial applications difficult. To improve the permselectivity of TFC membranes even further, various nanomaterials such as inorganic nanoparticles (NPs) and graphene oxide (GO) nanosheets were used [7]. Carbon nanotubes (CNTs) [8], porous metal–organic molecular cages, metal–organic frameworks (MOFs) [9], metal oxides [10], layered double hydroxides [11], and covalent organic frameworks [12], etc., were introduced into the PA layer. These nanomaterials improved the membrane hydrophilicity, augmented water transfer channels, and imparted anti-fouling or anti-bacterial properties to the membranes. These led to the swift growth of the so-called thin-film nanocomposite (TFN) membranes prepared by various strategies. 

Incorporating NPs into the TFC membrane during the IP process can be a promising approach as it improves the overall stability of these NPs, partially or fully encapsulated in the PA layer. Mass transfer resistance within the PA layer is reduced with the additional nanochannels and interfacial voids formed by the incorporation of nanoparticles in the selective layer of the composite membrane. Nonetheless, challenges such as weak adhesion between inorganic NPs and the active layer, non-uniform distribution, and NP aggregation pave the way for defects within the PA layer, which can have a major impact on the membrane’s physical stability and solute selectivity [13]. The lack of a robust TFN membrane with enhanced desalination performance and exceptional anti-fouling ability is a research gap that must be addressed. 

Among various NPs used so far for TFN membrane fabrication, mesoporous silica materials such as MCM-41 (Mobil Composition of Matter-41) [14] (pore size:15–100 Å) and SBA-15 (Santa Barbara Amorphous-15) [15] (pore size: 50–130 Å) have attracted considerable attention due to their excellent mechanical stability, biocompatibility, and surface hydrophilicity [16]. In this class of nanomaterials, MCF (Mesostructured Cellular Foam) silica is the least explored nanofiller for membrane modification. The unique properties of this siliceous material, such as large pore size (150–500 Å) and continuous three-dimensional (3D) pore system, can be utilized in fabricating TFN membranes. Functionalizing this MCF silica is also possible since the silica exhibits very similar chemical properties to the MCF-41 and SBA-15 [17]. To minimize particle agglomeration in the skin layer of TFN membranes, mesoporous materials can be functionalized with organic compounds and inorganic materials [18,19]. 

Polydopamine (PDA), a mimic of the adhesive foot proteins secreted by deep-sea mussels, is gaining popularity due to its increased hydrophilicity and surface adaptability [20]. PDA can form thick nanosized coatings on various surfaces, contributing to its wide application, including surface modification [21]. The PDA coating helps the NPs attach to the base membrane with excellent stability. Song et al. presented a simple and elegant method to fix silver in situ by exposing PDA to an AgNO_3_ solution in a recent study on synthesizing metal/organic hybrid nanomaterials. PDA’s reducing catechol groups immobilized silver ions, resulting in uniformly dispersed nanoparticles [22]. Zinc oxide (ZnO) nanoparticles have been gaining increasing attention in membrane separation due to their cost-efficient, antimicrobial properties; excellent affinity with water molecules; low toxicity; and anti-corrosive properties. With the aid of appropriate bio-glue materials, the dispersibility of metal oxides can be enhanced within porous silica carriers [23,24,25]. 

Zn/PDA/MCF-5 cellular foam structured composite with nanochannels arranged in order is a new category of three-dimensional foam material. Zn/PDA/MCF-5 cellular foam could improve physiochemical properties and filtration performance through a synergistic hydrophilic effect in their nanochannels. In this study, we propose anchoring zinc oxide onto the MCF-5 silica using the self-polymerizing PDA. PDA deposition can act as a green anchoring agent and aid in the in situ reduction of the metal salts to produce well-distributed zinc oxide nanoparticles [26]. The synthesized zinc oxide anchored siliceous mesostructured cellular foams (Zn-PDA-MCF-5) were encapsulated in the PA layer of the NF membrane for desalinating water. A series of characterizations were conducted to understand the effect of synthesized Zn-PDA-MCF-5 on TFN membranes’ physical and chemical properties. The result of different concentrations of Zn-PDA-MCF-5 on flux; rejection of monovalent, mono, or divalent salts; and 2,4-dichlorophenol removal was evaluated. The fouling tendency of the TFN membranes was studied using a model protein solution (bovine serum albumin), and the long-term filtration and the reproducibility of the membrane performance were studied.

## 2. Materials and Methods

### 2.1. Chemicals

Tetraethyl orthosilicate (TEOS, Sigma-Aldrich, 98.0%) as silica source, P123 (Poly(ethylene oxide)-block-poly (propylene oxide)-block-poly (ethylene oxide), EO20-PO70-EO20, Sigma-Aldrich, St. Louis, MO, USA; M_av_ = 5800) as a structure-directing agent, and 1,3,5 trimethyl benzene xylene (TMB) as the organic templating agent were utilized to synthesize siliceous mesostructured cellular foams (MCFs). Dopamine hydrochloride (Hi media, 98.0%), Tris-HCl (extra pure AR, Sisco Research Laboratories Pvt. Ltd., Mumbai, India, 99.0%), sodium hydroxide (NaOH, Spectrum Reagents Chemicals Pvt. Ltd., Ernakulam, India, 97.0%), hydrochloric acid (HCl, ≥35%, EMPLURA^®^ Merck, Mumbai, India), and zinc nitrate hexahydrate (Zn(NO_3_)_2_.6H_2_O, EMPLURA^®^ Merck, India ≥96.0%) were employed for the functionalization of the MCFs. Polyether sulfone (PES, granules, Sigma-Aldrich, MW = 58,000 g/mol), along with poly (ethylene glycol) (PEG, M_av_ = 400, Sigma-Aldrich, St. Louis, MO, USA) in N-methyl-2-pyrrolidone (NMP, Sisco Research Laboratories Pvt. Ltd. India, 99.5%), were used to fabricate the base membrane. M-phenylenediamine (MPD, flakes, Sigma-Aldrich, St. Louis, MO, USA; 99.0%), trimesoyl chloride (TMC, Aldrich, St. Louis, MO, USA; 7yhfc nghf4≥98.0%), and n-hexane (EMPLURA^®^ Merck, India ≥95.0%) were the monomers employed for the interfacial polymerization (IP) reaction. Inorganic salts–sodium chloride (NaCl, Merck, India ≥99.0%), sodium sulfate (Na_2_SO_4_, NICE, 99.0%, India), and magnesium chloride (MgCl_2_, NICE, 97.0%) were used to evaluate the membrane performance. The deionized (DI) water (18 MΩ·cm) obtained from a Milli-Q ultrapure water purification system (Elga pure lab Q-15) was used throughout the experiment. All the chemicals were used as received without any further purification.

### 2.2. Synthesis of Zinc Anchored Siliceous Mesostructured Cellular Foams (Zn-PDA-MCF-5)

MCF-5 was prepared following the previous protocol (Figure S1) [27]. Briefly, P123 (2.0 g, 0.4 mmol) was dissolved in 2 M HCl (75 mL) in a beaker covered with a watch glass, followed by the addition of TMB (2.0 g, 17 mmol), and the resultant mixture was heated to 40 ± 2 °C. The solution was stirred using a magnetic stirrer for 1 h, followed by the dropwise addition of TEOS. At the end of 20 h, the solution was transferred to an autoclave and aged at 100 °C for 24 h under static conditions. The mixture was then allowed to cool to ambient temperature. The white precipitate was separated using a filter paper, dried in air for 48 h, and finally calcined at 550 °C for 5 h in the air to combust the organic templates.

To obtain PDA-coated MCF-5, 50 mg of the synthesized siliceous MCF-5 was dispersed in a beaker containing 100 mL of 12.5 mM Tris-HCl solution (pH 8.5) with 25 mg of dopamine hydrochloride using an ultrasonic bath. After 0.75 h, the mixture was transferred to a shaker and incubated for 24 h at 25 °C. This solution was centrifuged at 4000 rpm for 0.5 h to separate the unreacted monomers, and the precipitate obtained was washed three times with ultrapure water and dried [28]. The obtained composite was denoted as PDA-MCF-5. In the final step, PDA-MCF-5 (35 mg) was added to a solution containing Zn(NO_3_)_2_·6H_2_O (8 mL, 32 mM) at 80 °C, pH = 8.5, and the resulting solution was stirred for 12 h at 80 °C. The precipitate obtained by centrifugation (4000 rpm for 0.25 h) was washed and dried to obtain a product denoted as Zn-PDA-MCF-5.

### 2.3. Preparation of TFC and TFN Membranes

The base membrane was prepared by the NIPS technique, wherein the polymer solution comprised 16 wt.% PES and 2.5 wt.% PEG-400 dissolved in NMP solvent. The solution was spread onto a clean glass substrate using a thin-film applicator (Elcometer 404), maintaining a thickness of 250 µm. The glass plate was immersed in the non-solvent (here, D.I. water) bath after 30 s to form the base membrane. The membrane was rinsed thoroughly to remove residual solvent and stored in deionized water until further use.

**Figure 1:**
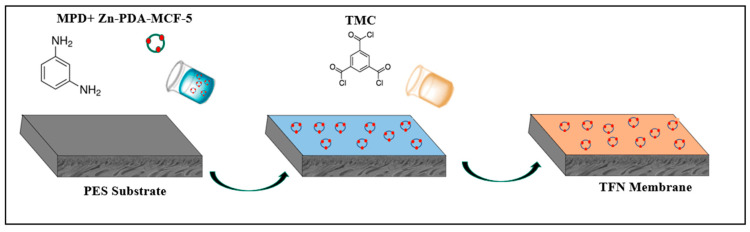
Schematic illustration of TFN membrane fabrication.

The in situ interfacial polymerization prepared the thin polyamide selective layer between 2.00 wt.% MPD (aqueous phase) and 0.15 wt.% TMC (n-hexane) (Figure 1). The MPD solution was poured over the top surface of the PES substrate and was held for 1 min, and the excess solution was removed with a rubber roller. The TMC solution was then introduced onto the surface for 1 min, and the solution was drained off. The membrane was then cured in an oven at 80 °C for 8 min, followed by rinsing with tap water to remove the residual monomers, and the obtained membrane, denoted as TFN-0, was stored in DI water.

To prepare TFN membranes, known concentrations (0.10, 0.25, 0.38, 0.50 wt.% with respect to the MPD concentration) of the Zn-PDA-MCF-5 material were dispersed into the aqueous phase. The subsequent fabrication steps and conditions remained unchanged, and the membranes were denoted as TFN-1, TFN-2, TFN-3, and TFN-4.

### 2.4. Characterization

The surface morphology analysis of the nanoparticle and membrane was carried out using a field-emission scanning electron microscope (FE-SEM) (SU6600 Analytical VP FE-SEM, Hitachi High Technologies America, Inc., Schaumburg, IL, USA). A high-resolution transmission electron microscope (HR-TEM) (JEM-3010 TEM, Joel Ltd., Tokyo, Japan) was used to study the internal structure of nanoparticles. The nanoparticles were dispersed in ethanol, placed onto the Cu grid, and dried before imaging. X-ray diffraction (XRD) patterns of nanoparticles and membranes were obtained using a Bruker D8 Advance-Twin-Twin diffractometer (Massachusetts, USA) with Cu Kα radiation (λ = 1.54 Å). The samples were scanned using a step size of 0.02 at a speed of 75.60 s per step in a continuous mode. Thermogravimetric analysis (TGA) of nanoparticles was carried out under a constant nitrogen flow at a 10 °C/min heating rate on the Hitachi STA7200, Japan TGA instrument. The chemical composition and functional groups of the nanoparticle and membrane were analyzed using dispersive energy X-ray (EDX) analysis (Joel 6390LA/OXFORD XMX N) and Fourier transform infrared spectroscopy (FTIR in transmission mode, Cary 630, Agilent Technologies, Santa Clara, CA, USA). The surface area of the nanoparticle was measured with a Brunauer–Emmett–Teller (BET) sorption instrument (Belsorp Max, Microtrac Belcorp, Japan) using liquid N_2_ at 77K. The membrane surface roughness was measured with an atomic force microscope (AFM) (WITec GmbH, Ulm, Germany) using the tapping mode in air for a scan area of 5 × 5 µm^2^. The wettability of the membrane surface by water (in air) was carried out by the contact angle goniometer (Kyowa, DMs-401, Japan) in the sessile-drop mode in at least 8 different spots of each membrane sample to ensure reliability. The thermal stability of all the fabricated membranes was analyzed using thermogravimetric analysis (TGA). The zeta potential of the nanoparticles was measured using the Microtrac Zeta check Particle charge reader (Germany).

The membrane pore size was calculated using polyethylene glycol (PEG) with different molecular weights ranging from 200 to 1000 Da. A feed concentration of 1000 ppm of PEGs at 6 bar was used to test the membranes. According to previous work, the PEG removal from the feed and permeate solution was determined using TOC (Shimadzu, TOC-L_CPH_, Tykyo, Japan). Accordingly, the membrane’s pore size in terms of Stokes radius was calculated as follows (Equation (1)) [29,30]:

(1) $$r_{p}=16.73\times 10^{-12}\times M^{0.557}$$

where *M* is the molecular weight of PEG at 90%, and *r_p_* is the Stokes radius.

### 2.5. Nanofiltration Performance Assessment

NF performance of the fabricated membranes was investigated using a crossflow filtration cell (Sterlitech Corporation, CF042D cell, Auburn, WA, USA) with an effective membrane area of 0.0042 m^2^, operated at 6 bar pressure and 25 °C (Figure S2). The composite membrane was pre-pressurized using deionized water for 1 h at a transmembrane pressure of 7.5 bar and 25 °C. Pure water; a 1000 ppm solution of three salts, namely, NaCl, Na_2_SO_4_, and MgCl_2_; and an aqueous solution containing 2,4-dichlorophenol (500 ppm feed solution) with pH = 7 were used as feeds to test the permeation and rejection performance of the membranes. The flux (J_w_) and rejection (R) were calculated by Equations (2) and (3), respectively [7].

(2) $$J_{w}=\frac{V}{A\times \Delta t}$$

where J_w_ is the water flux (Lm^−2^ h^−1^, abbreviated as LMH), V is the volume of the permeate (L), A is the effective membrane area (m^2^), and Δt is the filtration time (h).

(3) $$R\left(\%\right)=\left(1-\frac{C_{P}}{C_{F}}\right)\times 100$$

C_f_ and C_p_ refer to the solute concentration in the feed and permeate, respectively. The salt concentration was calculated according to the standard curve between the concentration and the conductivity measured for each salt by a conductivity meter (Systronic conductivity meter 304). The rejection of 2,4-dichlorophenol (500 ppm feed solution) was determined using Equation (3) and quantified using a TOC Analyzer (Shimadzu TOC-L_CPH_, Japan). All experiments are repeated for three different membrane samples of the same composition (n = 3).

### 2.6. Evaluation of Antifouling Property 

The antifouling properties of both TFN-0 and TFN-2 membranes were evaluated through a crossflow filtration experiment with an aqueous solution containing BSA, which was used as a model foulant mimicking natural proteins present in wastewater. The fouling tests were conducted in the recirculation mode at 25 °C and pH 7.0 ± 0.1. Before the test, the membranes were pre-compacted for 1 h, and a stable pure water flux ($J_{w1}$) of these membranes was measured for about 1 h under the operating pressure of 6 bar. Then, the pure water was replaced by the foulant feed solution (500 ppm) for a 1 h test to measure the flux (J_P_). After 1 h, the fouled membranes were rinsed with deionized water. Lastly, the pure water flux ($J_{w2})$ of the washed membranes was measured. The indexes to judge the antifouling property were listed as follows (Equations (4) and (5)) [31]:

(4) $$\mathrm{FRR}\;(\%)=\left(\frac{J_{w2}}{J_{w1}}\right)\times 100$$

(5) $${\mathrm{DR}}_{t}\;(\%)=\left(1-\frac{J_{P}}{J_{w1}}\right)\times 100$$

(6) $${\mathrm{DR}}_{r}(\%)=\left(\frac{J_{w2}-J_{P}}{J_{w1}}\right)\times 100$$

(7) $${\mathrm{DR}}_{\mathrm{ir}}(\%)=\left(\frac{J_{w1}-J_{w2}}{J_{w1}}\right)\times 100$$

DR_t_, DR_r_, and DR_ir_ denote the total, reversible, and irreversible flux decline ratios, respectively. FRR denotes the flux recovery ratio, DR_r_ represents the fouling caused by the concentration polarization on the membrane surface, and DR_ir_ indicates the fouling caused by the BSA molecules due to their stable adhesion on the surface of the membrane. 

## 3. Results

### 3.1. Characterization of Mesoporous Cellular Foam Nanoparticles

**Figure 2:**
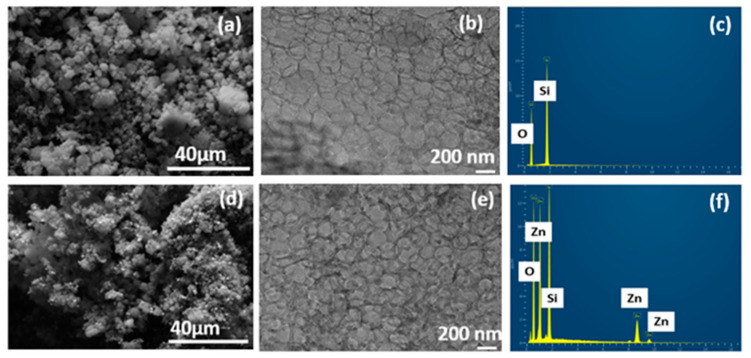
(**a**,**d**) FE-SEM micrograph; (**b**,**e**) HR-TEM image; (**c**,**f**) EDX spectra of MCF-5 silica and Zn-PDA-MCF-5.

The surface morphology of the synthesized siliceous MCF-5 and Zn-PDA-MCF-5 was imaged using the FE-SEM, as seen in Figure 2a,d. The results from FE-SEM analysis in Figure 2a reveal that the MCF-5 silica particles were entirely spherical morphologies with sizes up to a few micrometers, as evidenced by other researchers [32]. The PDA coating and ZnO anchoring made the surface slightly irregular (Figure 2d) without jeopardizing the mesopores [33].

The cellular foam structure, typical of MCF-5, was observed in the HR-TEM images (Figure 2b,e). The images were analyzed using ImageJ software, and the average cell diameter was 25.26 ± 4.09 nm. Both samples possessed a disordered array of silica struts, which is the characteristic structural feature of the MCFs. The PDA coating was also evident as fine deposition on and within the spherical cells, and the presence of ZnO can be noted as dark spots uniformly dispersed on the MCF-5 (Figure 2e). This finding is consistent with the reduction in pore volume encountered in the BET analysis. The EDX spectra of the pristine MCF-5 showed the presence of Si and O (Figure 2c) as expected, and the Zn-PDA-MCF-5 exhibited ≈14% (atomic %) Zn element additionally (Figure 2f, Table S1). 

**Figure 3:**
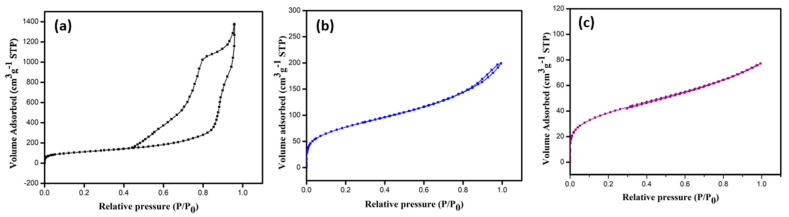
BET isotherms of N_2_ adsorption–desorption of (**a**) MCF-5 silica, (**b**) PDA-MCF-5, and (**c**) Zn-PDA-MCF-5.

Figure 3a–c shows the BET adsorption isotherms of the nitrogen (N_2_) adsorption and desorption at 77 K, and the corresponding pore size distribution curves are shown in Figure S3a–c. The pristine MCF-5 silica exhibited a type IV isotherm with an H3 hysteresis loop at a pressure > 0.42P_0_, characteristic of typical mesoporous material, consistent with the report on biomaterial-supported copper nanoparticles [34]. The modifications of the MCF-5 silica significantly affected the nitrogen uptake and are evident in Figure 3b,c. The PDA-MCF-5 and Zn-PDA-MCF-5 exhibited a type II isotherm. The specific surface area was calculated using the Brunauer–Emmett–Teller (BET) equation and adsorption data with relative partial pressures ranging from 0.05 to 0.5. The total pore volume was calculated using the BET plot and the amount of N_2_ at a relative partial pressure of 0.99. The nonlocal density function theory (NLDFT) was used to calculate the pore size distribution (PSD). The appreciable reduction in specific surface area, pore size, and pore volume (Table S2) of the modified materials (PDA-MCF-5 and Zn-PDA-MCF-5) demonstrate that the polydopamine coating was successful, and zinc anchoring was achieved [35].

**Figure 4:**
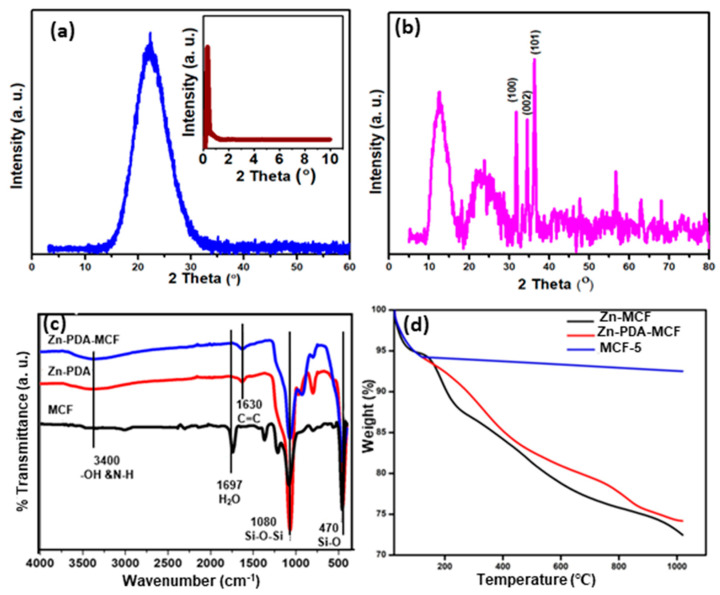
Wide-angle XRD of (**a**) MCF-5 silica and small-angle X-ray diffraction of MCF-5 silica (inset); (**b**) wide-angle XRD of Zn-PDA-MCF-5; (**c**) FT-IR spectra of mesoporous fillers; and (**d**) TGA curves for MCF-5, PDA-MCF-5, and Zn-PDA-MCF-5.

Figure 4a shows the XRD pattern of MCF-5 silica in the 2θ range of 3–60^0^. A broad diffraction peak between 10 and 40°, with a maximum at 2θ = 22.64°, is the typical amorphous silica diffraction peak [36]. The small-angle XRD (SA-XRD) pattern of synthesized MCF-5 silica is displayed in the inset of Figure 4a. It showed a diffraction peak in the range of 2θ = 0.30–2.00°, indicating the presence of a well-ordered structure and a narrow 3D spherical pore size distribution, in agreeance with the BET result [37]. The presence of zinc on the surface of PDA-MCF-5 was confirmed through the XRD analysis of Zn-PDA-MCF-5 (Figure 4b). The peak at 2θ values of 31.70, 34.40, and 36.30° corresponded to (100), (002), and (101) crystal planes, respectively, which are in significant agreement with standard JCPDS no 36-1451 [38]. This confirmed the existence of Zn in the form of ZnO on the PDA-MCF-5 surface. Moreover, the intensity of the diffraction peaks for the Zn-PDA-MCF-5 were relatively low as the peaks broadened, confirming the composite formation [39].

Figure 4c displays the FT-IR spectra of the synthesized siliceous MCF-5, PDA-MCF-5, and Zn-PDA-MCF-5 from 4000 cm^−1^ to 400 cm^−1^. The siliceous MCFs exhibited intense peaks at about 1080 cm^−1^ and 800 cm^−1,^ corresponding to the asymmetric and symmetric stretching vibrations of the Si-O-Si, respectively, in the framework, and the peak at 470 cm^−1^ can be ascribed to the bending vibration of the Si-O bond [40]. The weak broad peak between 3000 and 3400 cm^−1^ was attributed to stretching vibrations of the O-H bond. The PDA coating introduced a new peak centered at 1630 cm^−1^, which can be assigned to the stretching vibrations of the aromatic ring of polydopamine. The peak broadening at ≈3400 cm^−1^ can be explained by the combination of stretching vibration of the O-H and N-H groups [41]. A red shift was observed in all of the characteristic peaks of Zn-PDA-MCF-5, which could be explained by the interaction between the catechols (O-H) in polydopamine and the silica surface through hydrogen bonding, as well as by electrostatic interactions between the amine group in dopamine and zinc oxide nanoparticles [42,43] (refer to Figure S1).

The thermogram of the synthesized MCF-5 silica (Figure 4d) consisted of a two-stage mass loss. The first one, from 27 to 120 °C, corresponded to the local elimination of water molecules interacting with the surface silanol groups. At the temperature range of 120–1000 °C, a weight loss of about 2% was observed. The latter stage can be attributed to the dihydroxylation of the surface Si-OH groups [27]. The TGA curve of the PDA-MCF-5 and Zn-PDA-MCF-5 showed a mass loss of about 16% in the range of 180–800 °C, which was attributed to the decomposition of the PDA [44]. This was an indication of the successful coating of MCF silica with PDA.

### 3.2. Characterization of the Fabricated Membranes 

The chemical structure of the membranes was studied from the ATR-FTIR spectroscopy, as displayed in Figure S4a. The various distinct and unique peaks of the PES base membrane appeared in all the spectra, implying that the selective polyamide layer’s thickness was <1 µm [45]. The typical absorption bands at 1236 and 1147 cm^−1^ can be regarded as the asymmetrical and symmetrical stretching vibrations of the sulfone group, the absorption bands at 1578 cm^−1^ were associated with the benzene ring stretching vibrations, and one at 1485 cm^−1^ was attributed to the C-C stretching vibrations of the PES base membrane [46]. 

The coating of the PA layer was confirmed in both TFC and TFN membranes by the presence of peaks around 1660 and 1548 cm^−1^ corresponding to the amide I (C=O) and amide II (N-H) vibrations, respectively [47]. The peak at around 1080 cm^−1^ could be attributed to the asymmetric stretching vibration of Si-O-Si, indicating the successful incorporation of Zn-PDA-MCF-5 nanoparticles into the polyamide layer, which was evident for all TFN membranes.

The thermogravimetric analysis (TGA) of the prepared membranes in the temperature range of 27–1000 °C is shown in Figure S4b. The TGA test is widely employed to evaluate and assess the temperature stability of membranes. Weight loss occurred in two stages for all the membranes. The first one can be associated with the loss of adsorbed moisture and solvent residue [48]. Weight loss at temperatures ranging from 430 to 600 °C is caused by the decomposition of the PES backbone [49]. Patterns of weight loss of the TFNs were similar, indicating that the nanoparticles were uniformly distributed on the PA layer and did not cause any thermal instability.

**Figure 5:**
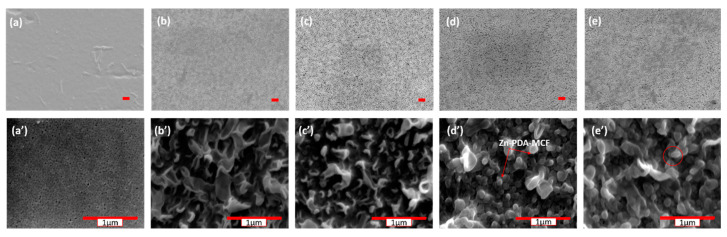
FE-SEM images of membranes—(**a**,**a′**) PES, (**b**,**b′**) TFN-0, (**c**,**c′**) TFN-1, (**d**,**d′**) TFN-2, and (**e**,**e′**) TFN-4. The marked scale (red) represents 1 µm, top images ×5000 and bottom ×50,000.

The SEM images of the top surface of the PES base membrane (Figure 5a,a′) showed a dense, smooth surface. However, TFC and TFN membranes that formed the polyamide layer after interfacial polymerization exhibited a leaf-like morphology. The surface morphology of the TFN-1 membrane was indistinguishable from the TFN-0 membrane. The Zn-PDA-MCF-5 present in the MPD solution (at higher concentrations) were able to provide additional surfaces for the IP reaction, increasing the reaction zone’s size. This led to forming leaf-like projections or “stipules” on the TFN membrane surface instead of the leaf-like morphology [50].

Further, the stipules formed due to the greater concentration of nanoparticles were marked in a red circle. Figure S5 illustrates the cross-sectional morphology profile of TFC and nanomodified TFN membranes. The total thickness of the membranes was around 150 ± 14 µm. Nanoparticles added to TFC membranes altered the thickness of the skin layer. TFN showed a higher thickness than TFC. The thickness of the selective polyamide layer was estimated using ImageJ software and was found to be 250–350 nm. This was because porous nanoparticles may provide adequate space for monomers to migrate for interfacial polymerization, thus increasing membrane thickness. 

**Figure 6:**
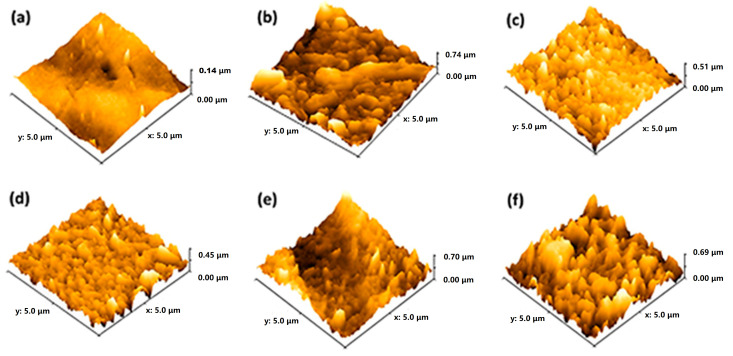
Surface roughness using AFM imaging of (**a**) PES, (**b**) TFN-0, (**c**) TFN-1, (**d**) TFN-2, (**e**) TFN-3, and (**f**) TFN-4.

The roughness parameters of the TFN-2 membrane were found to be much lower when compared to all the fabricated composite membranes (Figure 6a–f, Table S3). The reduction in surface roughness can be explained as the formation of a monolayer (PA) up to an optimal loading of the nanoparticles [51]. It is evident from the AFM images that TFN-4 possessed rougher surface morphology due to the agglomeration of the nanoparticles, consistent with the SEM images. Figure S6 displays the molecular weight cut-off (MWCO) of TFN-0 and TFN-2 membranes. The MWCOs of TFN-0 and TFN-2 were 489 (or 0.53 nm) and 365 (or 0.45 nm), respectively, corresponding to the 90% PEG rejection. The nanoparticle contained pores with oxygen/nitrogen-containing functional groups that enhanced the interfacial polymerization reaction (IP) by not obstructing the migration of monomers during IP. Similar observations were found in previous studies. In addition, the increasing trend in membrane thickness was consistent with the findings of MWCO (refer to SEM results, Figure S5). 

The dynamic water contact angle (WCA) recorded for 60 s was used to assess the wettability of the fabricated membranes. Figure S7 demonstrates the increased membrane hydrophilic character in incorporating the Zn-PDA-MCF-5. The water contact angles of the PES base membrane and TFN-0 were estimated at 68.2 ± 2.9° and 60.4 ± 3.1°, respectively. The PES base membrane’s limited wettability was due to the polyether sulfone polymer’s intrinsic hydrophobicity. The hydrophilic PA layer further improved the surface wettability [52], and at the optimum concentration of Zn-PDA-MCF-5 (for TFN-2), the WCA was the lowest and showed a rapid decline (rapid spreading). The decrease in the WCA of the TFN-2 and TFN-3 membranes can be accounted in terms of the presence of large amounts of hydroxyl and amine functional groups on the filler surface, playing an essential role in forming a hydration layer with water via hydrogen bonding [35]. The formation of carboxylic acid functional groups produced by the hydrolysis of residual acyl chloride groups can also explain the enhanced surface wettability [53]. It was observed that TFN-4 displayed a considerable increase in water contact angle due to the surface roughness caused by the aggregation of nanoparticles; however, it showed greater hydrophilicity compared to the TFN-0 membrane [54]. The increase in WCA can be explained as follows: at higher concentrations, the aggregation of the nanoparticles occurs to minimize their surface free energy, which leads to their uneven distribution within the polyamide layer. This can prevent the surface wetting of the membrane, leading to greater contact angle values [55].

### 3.3. The Performance of TFN Membranes

#### 3.3.1. Pure Water Permeability (PWP)

**Figure 7:**
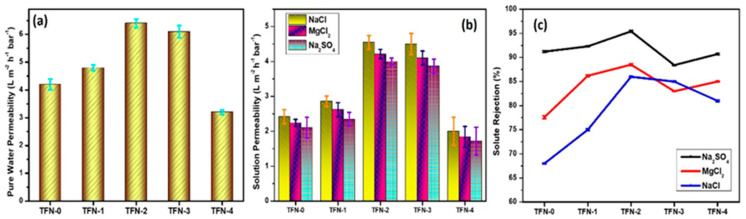
Filtration performance of the membranes: (**a**) pure water permeability; (**b**) solution permeability; and (**c**) solute rejection (experimental conditions: pressure—6 bars, feed concentration—1000 ppm, pH—7.00, membrane area = 0.0042 m^2^, n = 3).

The PES base membrane exhibited a PWP of 15.125 ± 2.03 LMH bar^−1^; however, the TFC membranes exhibited a much lower flux, owing to the highly crosslinked PA layer. The hydrophilic nature of nanoparticles embedded in the PA layer improves water molecule permeation through membranes [56]. The TFN-2 membrane exhibited the highest PWP of 6.4 ± 0.15 LMH bar^−1^, 35% greater than the TFN-0 (Figure 7a). As evidenced by the WCA studies, the membrane’s enhanced surface hydrophilicity indicated relatively easy surface wetting and absorption of water molecules on the membrane surface, which helps to improve water permeability [57]. Moreover, the interaction of the membrane matrix with nanoparticles can positively influence the geometry of the membrane pores to improve the permeation properties of the membrane. 

Furthermore, the mesoporous windows, characteristic of incorporated Zn-PDA-MCF-5 nanoparticles, could provide extra added short water permeation pathways through the PA layer, similar to the reports for TFN membranes containing graphene oxide (GO) nanosheets in the active layer [58]. Higher concentrations of Zn-PDA-MCF-5 (0.5 wt.%) in the PA layer could have an uneven distribution, resulting in a partially compact surface structure and can also form a thicker PA layer (Figure S5). Combined with the decreased hydrophilicity, the pure water permeability of TFN-4 membranes decreased distinctly [59].

#### 3.3.2. Desalination Performance of the Membranes

The solution permeability and salt rejection were evaluated from individual synthetic solutions containing divalent and monovalent inorganic salts (Na_2_SO_4_, MgCl_2_, NaCl), and the results are provided in Figure 7b,c. The TFN-0 has an isoelectric pH of 3.5, above which the membrane possesses a negative charge [60]. The residual carboxyl groups and the introduced negatively charged nanoparticles endowed the surface of the PA layer with negative charges. The zeta potential of MCF-5 and Zn-PDA-MCF-5 were found to be −70 ± 1.80 mV and −66 ± 2.10 mV, respectively. The slight decrease in negative charge could be attributed to the amine functional groups over PDA [61]. This indicates that the TFN membranes would have a greater negative surface charge than the TFN-0 at the experiment pH of 7 [62,63].

The solution permeability of the membranes showed a similar trend to the PWP study (Figure 7b), i.e., the solution permeability increased as the Zn-PDA-MCF-5 loading increased up to TFN-3 but declined with further loading. All membranes displayed a similar rejection pattern: R(Na_2_SO_4_) > R(MgCl_2_) > R(NaCl), which was in good agreement with the separation characteristics of a negatively charged NF membrane [64]. The synergistic effect of Donnan exclusion and size sieving can be applied to explain the observed rejection trend [65]. The electrostatic interaction between the ions in the feed solution and the membrane significantly impacted the salt rejection for a charged membrane [66].

The Donnan exclusion effect states that negatively charged groups on the membrane surface can attract a high-valent cation while repelling a high-valent anion, resulting in a high rejection of salts containing a high-valent anion. It was observed that the negatively charged TFN membranes exhibit a higher rejection of the divalent anion (SO_4_^2−^) compared to the monovalent anion (Cl^−^), which could be explained by the stronger electrostatic repulsion and the lower diffusion coefficient of SO_4_^2−^ [7] (Table S4). The rejection of MgCl_2_ was greater than NaCl due to the larger hydration radius and lower diffusion coefficient of Mg^2+^ than Na^+^ ions. Additionally, when exposed to operational pressure, Na+ ions can separate from their hydration shells and diffuse through the membranes, resulting in lower rejection. At the same time, the strong hydration shell of Mg^2+^ restricted their diffusion through the membrane and was thus rejected [67].

The variation in permeability and salt rejection strongly relied on the shear plane and surface of NF membranes, which were influenced by the type of salt used, nanoparticle loading, and solution conditions [68]. Table 1 shows a comparison of this study to the most recent literature. TFN-2, the best-performing membrane, demonstrated excellent rejection of both divalent and monovalent salts with comparable pure water flux, making it a suitable candidate for further testing.

#### 3.3.3. Long-Term Salt Filtration

**Figure 8:**
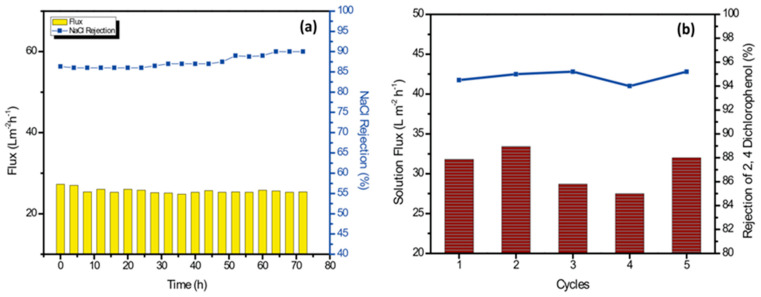
(**a**) Long-term performance of the TFN-2 membrane for filtration of 1000 ppm NaCl solution. (**b**) Solution flux and rejection studies with 2,4-dichlorophenol (500 ppm solution) for 5 cycles.

As shown in Figure 8a, the operational stability of the TFN-2 membrane was evaluated for the filtration of 1000 ppm sodium chloride solution for 72 h. The TFN-2 membranes were robust and exhibited no/minimal change in permeate flux and salt rejection. The NaCl rejection gradually increased from ≈86% to >90% after 72 h of operation with a slight loss of membrane permeability, which can be attributed to the membrane compaction. Overall, the Zn-PDA-MCF-5-incorporated PA layer was stable for long-term desalination performance.

#### 3.3.4. Removal of Organic Micropollutants and Anti-Fouling Performances

Chlorinated phenols are toxic micropollutants present in wastewater from pesticide manufacturing, paper manufacturing, oil refinery wastewater, etc. They are also formed during the chlorination of municipal wastewater [75]. The efficacy of TFN-2 for removing a model pollutant, 2,4-dichlorophenol, was investigated using the TFN-2 membrane (Figure 8b). There were no significant changes in water permeability or solute rejection for up to five operation cycles. The TFN-2 membrane displayed an average rejection of 94.78 ± 0.5% and a water flux of 30.30 ± 2.45 Lm^−2^ h^−1^. 

Water washing was able to easily remove the organic contaminant bound to the membrane surface during the run. This could have been because the 2,4-dichlorophenol contains chloride ions on the aromatic ring, thus forming a more stable anionic oxygen atom within the hydroxyl group. The negatively charged membrane repelled this negatively charged pollutant. Further, the membrane hydrophilicity and lower surface roughness prevented the adsorption of the organic pollutant. On the basis of these results, it can be inferred that the Zn-PDA-MCF-5 incorporated TFN membrane produced stable separation performances. 

**Figure 9:**
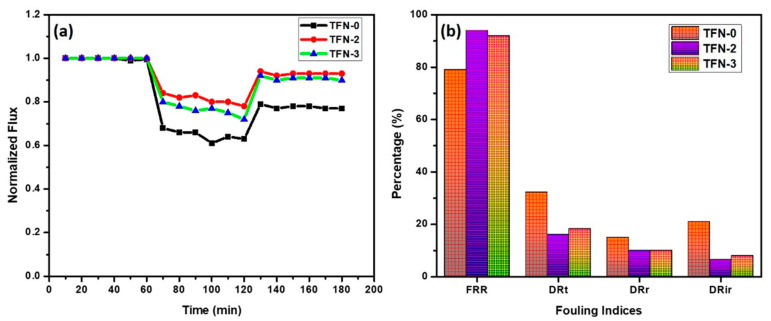
(**a**) Anti-fouling study and (**b**) fouling indices of the prepared membranes.

Membranes with excellent anti-fouling properties would reduce the frequency of chemical cleaning or water backwash, thus improving the lifespan of the membrane [76]. An integrated flux profile and a summary of the anti-fouling indexes of TFN-0, TFN-2, and TFN-3 membranes are presented in Figure 9a,b. It was observed that the introduction of Zn-PDA-MCF-5 nanoparticles enhanced the anti-fouling performance of the membranes when challenged with a model protein foulant, BSA. The DRt and FRR of the pristine TFN-0 membrane were 32.22% and 78.98%, respectively. In TFN-2 membranes, where Zn-PDA-MCF-5 nanoparticles are embedded in the polyamide layer, DRt decreased to 16.14%, and the FRR increased to 94.00%. The lower DRt value indicated the improved anti-fouling property of the TFN-2 membrane, while the higher FRR value implied that the model foulant adsorbed on the surface of the TFN-2 membrane could be more readily washed off by simple hydraulic washing [77]. The increased FRR of TFN-2 was harmonized with its water contact angle. The irreversible fouling ratio (DRir) decreased from 21.01% for pristine TFN-0 membranes to 6.54% for TFN-2, showing an improvement in surface fouling resistance due to the presence of Zn-PDA-MCF-5 particles. The dramatic reduction in time-dependent flux observed after replacing the deionized water with the BSA solution was caused by the accumulation of foulant on the membrane surface, which led to concentration polarization and membrane fouling [78].

Membrane fouling can be mitigated by reducing the adsorptive interactions between foulant and the membrane surface, including Van der Waals attractions, hydrogen bonding, and hydrophobic/electrostatic interactions [79]. The better anti-fouling performance of TFN-2 membranes can be attrivuted to their higher hydrophilicity, lower surface roughness, and negative surface charge. Increased hydrophilicity of the TFN-2 membranes results in the adsorption of water molecules on the membrane surface and the formation of a hydrated layer that can effectively alleviate the hydrophobic interactions between foulant molecules and the membrane surface. This could have been the main reason for the improved flux recovery ratio of the modified membrane to the model foulant of BSA. On the other hand, the superior anti-fouling properties (lower irreversible fouling) could be attributed to the electrostatic repulsion between the negatively charged BSA (at neutral pH) and the TFN-2 membrane [31].

## 4. Conclusions

Thin-film nanocomposite membranes were fabricated, incorporating a novel nanomaterial—Zn-PDA-MCF-5—in the polyamide selective layer. Incorporating the hydrophilic modifier improved membrane hydrophilicity, lowered surface roughness, and enhanced filtration capabilities. The improved membrane permeability (TFN-2) was due to its lower WCA, smoother surface, and the nanoparticles providing additional flow channels or interconnected short flow paths.

The solution permeability was in agreement with the pure water permeability, and the rejection of salts followed the order R(Na_2_SO_4_) > R(MgCl_2_) > R(NaCl), which could be explained by the size sieving and Donnan exclusion mechanisms. The best membrane TFN-2 displayed a PWP of 6.4 LMH bar^−1^ and a rejection of 95.48 ± 0.20/88.34 ± 0.15/86.34 ± 0.22% for Na_2_SO_4_/MgCl_2_/NaCl, respectively. The membranes exhibited stable flux and rejection for NaCl for over 96 h when evaluated with a synthetic feed solution. The membrane also showed excellent rejection (>95%) for a model organic pollutant 2,4-dichlorophenol with excellent stability for more than five cycles. The negative surface charge, lower surface roughness, and enhanced hydrophilicity could explain the high flux recovery ratio (>94%) and low irreversible fouling when challenged with model foulant BSA. The fabricated membranes hold great potential to be further investigated with real effluents.

## Figures and Tables

**Table 1:** A comparison of the performances of the TFN membranes.

Base Polymer	Filler Used	Pure Water Permeability (LMH bar^−1^)	Salt Rejection (%)	Ref.
PSf	GO	2.43	Na_2_SO_4_—95.20 MgCl_2_—62.10 NaCl—59.50	[69]
PSU	GO	2.87	Na_2_SO_4_—97.30 ± 0.30 NaCl—93.80 ± 0.60	[57]
PES	GO-COCl	4.80	Na_2_SO_4_—86.90	[70]
PSf	SCQD	5.30	Na_2_SO_4_—93.10	[64]
PSf	Amine-functionalized GO	12.40	Na_2_SO_4_—98.20 MgCl_2_—93.40 NaCl—38.20	[7]
PES	Boron nitride nanosheets	7.65	Na_2_SO_4_—88.30 ± 30 NaCl—12.60 ± 0.80	[71]
PES	ssDNA	12.63	Na_2_SO_4_—98.00 MgCl_2_—72.80 NaCl—23.00	[72]
PSf	PDA-SiNPs	13.33	Na_2_SO_4_—97.00 MgCl_2_—68.00 NaCl—35.00	[73]
PSf	CNC	2.25	Na_2_SO_4_—93.44 MgCl_2_—79.52 NaCl—62.68	[74]
PES	Zn-PDA-MCF-5	6.40 ± 0.15	Na_2_SO_4_—95.48 ± 0. 20 MgCl_2_—88.34 ± 0.15 NaCl—86.34 ± 0.22	This work

## Data Availability

Data are contained within the article.

## Supplementary Materials

The following supporting information can be downloaded at https://www.mdpi.com/article/10.3390/membranes13050486/s1, Figure S1. Schematic illustration of synthesis of Zn-PDA-MCF-5 nanoparticle. Figure S2. Schematic illustration of crossflow NF system. Figure S3. Pore size distribution of (a) MCF-5 silica (b) PDA-MCF-5 (c) Zn-PDA. Figure S4. (a) FT-IR spectra and (b) TGA Curves of fabricated membranes. Figure S5. Cross-sectional morphologies of (a, a’) PES; (b, b’) TFN-0; (c, c’) TFN-2; and (d, d’) TFN-4 membranes. Figure S6. Molecular weight cut-off of membranes. Figure S7. Dynamic water contact angles of the membranes. Table S1. Elemental composition from nanoparticles. Table S2. Brief summary of surface areas and pore sizes of mesoporous fillers. Table S3. Roughness parameters of the membranes from AFM. Table S4. Parameters of inorganic ions in the feed solution [64,80].
