# Supplementary material for: ZnO/PDA/Mesoporous Cellular Foam Functionalized Thin-Film Nanocomposite Membrane towards Enhanced Nanofiltration Performance
Source: Membranes (Basel). 2023 Apr 29;13(5):486. doi: 10.3390/membranes13050486 (PMC10222560; doi:10.3390/membranes13050486)
Supplement: Supplementary file 1 [file membranes-13-00486-s001.zip › membranes-2353694-supplementary.pdf]

membranes

MDPI

Article

ZnO/PDA/Mesoporous Cellular Foam Functionalized Thin-Film Nanocomposite Membrane towards Enhanced Nanofiltration Performance

Jenny Nambikkattu [1], Anoopa Ann Thomas [1], Noel Jacob Kaleekkal [1,*], Thanigaivelan Arumugham [2,3], Shadi W. Hasan [2,3] and Saravanamuthu Vigneswaran [4,5,*]

1 Membrane Separation Group, Department of Chemical Engineering, National Institute of Technology Calicut (NITC), Kozhikode 673601, India
2 Department of Chemical Engineering, Khalifa University, Abu Dhabi P.O. Box 127788, United Arab Emirates
3 Center for Membranes and Advanced Water Technology (CMAT), Khalifa University of Science and Technology, Abu Dhabi P.O. Box 127788, United Arab Emirates
4 Centre for Technology in Water and Wastewater, School of Civil and Environmental Engineering, University of Technology Sydney, Sydney, NSW 2007, Australia
5 Faculty of Sciences &amp, Technology (RealTek), Norwegian University of Life Sciences, P.O. Box 5003, 1432 As, Norway
* Correspondence: noeljacob89@gmail.com or noel@nitc.ac.in (N.J.K.); saravanamuth.vigneswaran@uts.edu.au (S.V.)

Citation: Nambikkattu, J.; Thomas, A.A.; Kaleekkal, N.J.; Arumugham, T.; Hasan, S.W.; Vigneswaran, S. ZnO/PDA/Mesoporous Cellular Foam Functionalized Thin-Film Nanocomposite Membrane Towards Enhanced Nanofiltration Performance. *Membranes* **2023**, *13*, 486. https://doi.org/10.3390/membranes13050486

Academic Editor: Benjamin S. Hsiao

Received: 03 April 2023
Revised: 21 April 2023
Accepted: 26 April 2023
Published: 29 April 2023

Copyright: © 2023 by the author. Licensee MDPI, Basel, Switzerland. This article is an open access article distributed under the terms and conditions of the Creative Commons Attribution (CC BY) license (https://creativecommons.org/licenses/by/4.0/).

Supplementary Information

Figures

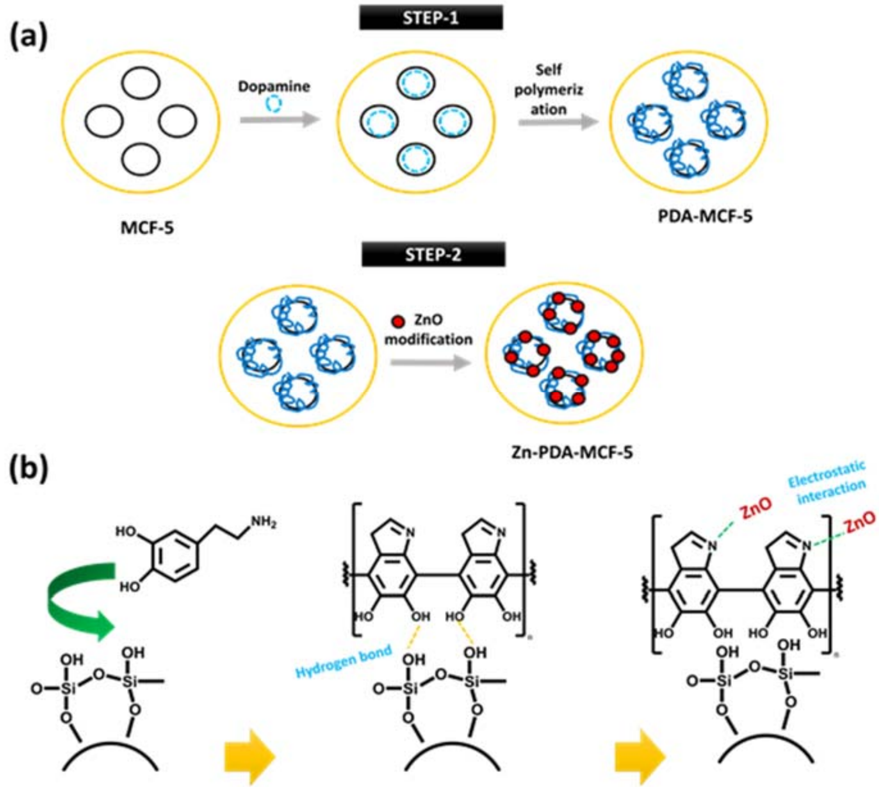

Figure S1. Schematic illustration of synthesis of Zn-PDA-MCF-5 nanoparticle.

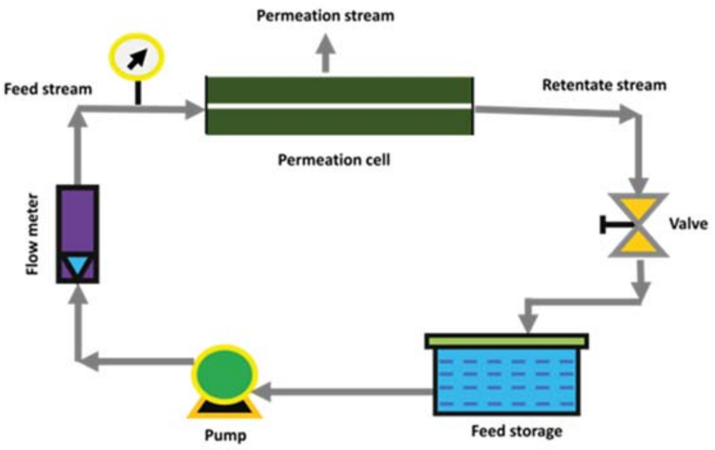

Figure S2. Schematic illustration of crossflow NF system.

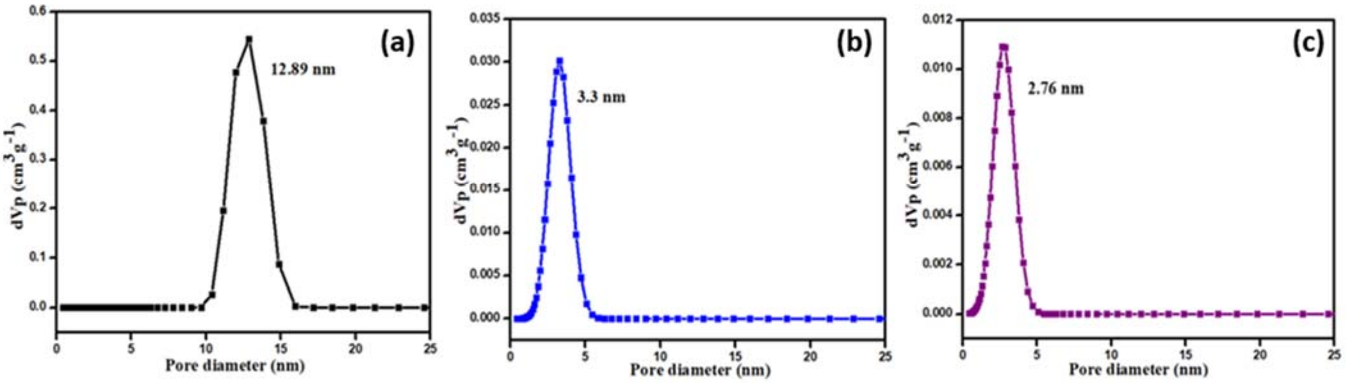

Figure S3. Pore size distribution of (a) MCF-5 silica (b) PDA-MCF-5 (c) Zn-PDA.

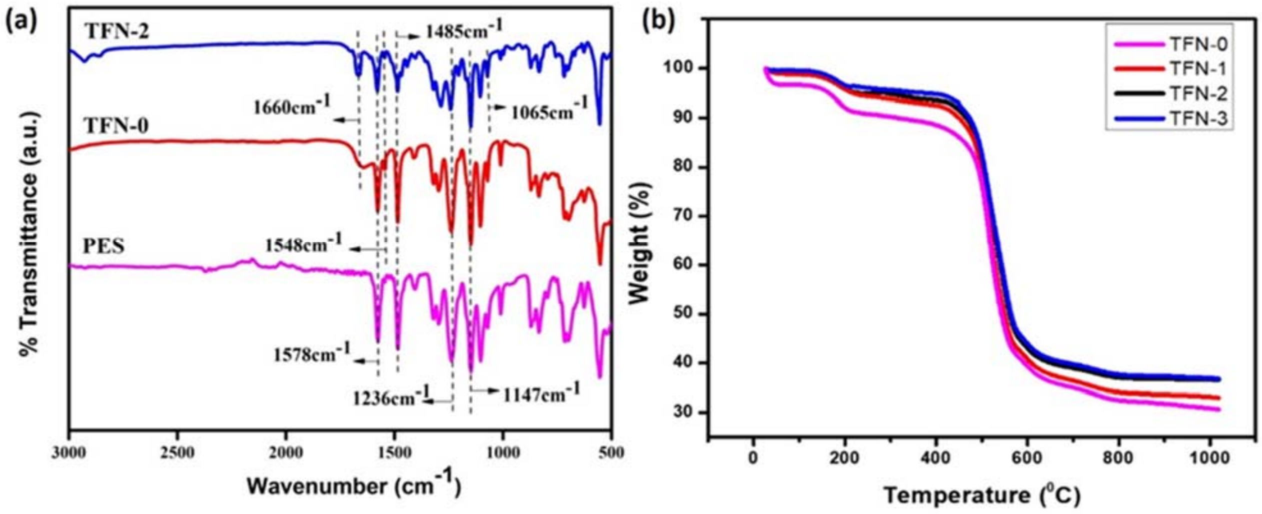

Figure S4. (a) FT-IR spectra and (b) TGA Curves of fabricated membranes.

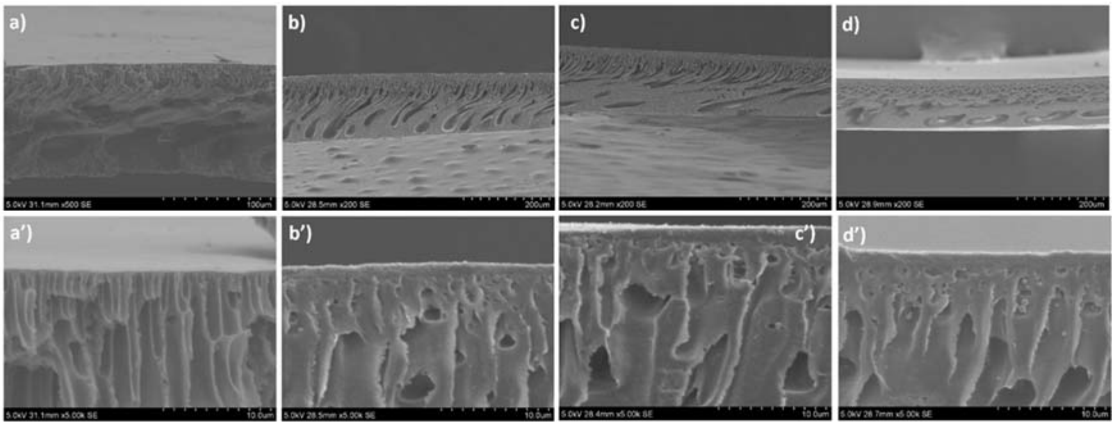

Figure S5. Cross-sectional morphologies of (a, a') PES; (b, b') TFN-0; (c, c') TFN-2; and (d, d') TFN-4 membranes.

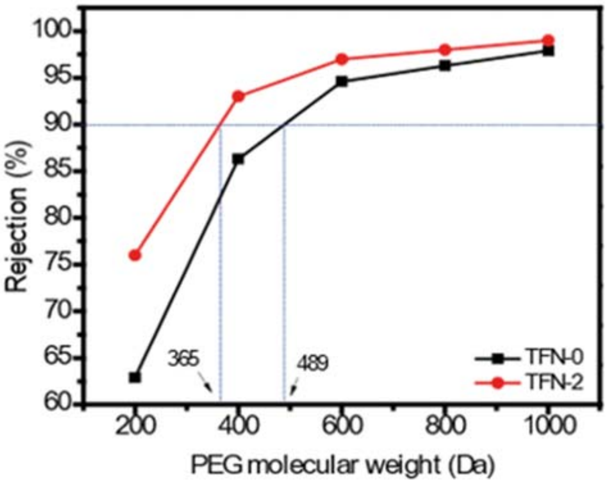

Figure S6. Molecular weight cut-off of membranes.

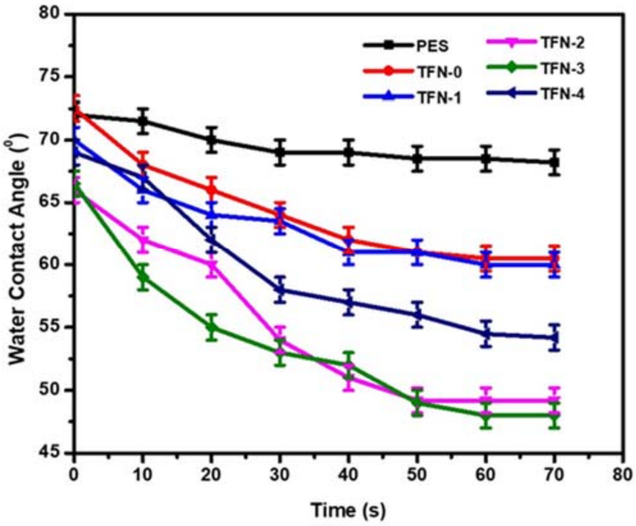

Figure S7. Dynamic water contact angles of the membranes.

Tables

Table S1. Elemental composition from nanoparticles.

Element	Atomic wt.%	
	MCF silica	Zn-PDA-MCF-5
O	72.25	52.50
Si	27.75	10.55
C	0	18.52
N	0	4.67
Zn	0	13.76

Table S2. Brief summary of surface areas and pore sizes of mesoporous fillers.

Samples	Specific Surface Area[a] (m^2/g)	Pore Volume[a] (cm^3/g)	Pore Size[b] (nm)
MCF-5	389.71	2.13	12.89
PDA-MCF-5	278.12	0.30	03.29
Zn-PDA-MCF-5	138.92	0.12	02.76

[a] Calculation based on BET method. [b] Calculation based on NLDFT method.

Table S3. Roughness parameters of the membranes from AFM.

Membrane ID	Ra (nm)	Rz (nm)	Rrms (nm)
**PES**	12.80 ± 1.70	40.20 ± 6.30	15.60 ±1.90
**TFN-0**	47.80 ± 12.60	133.30 ± 26.90	59.50 ± 19.30
**TFN-1**	45.20 ± 7.70	164.80 ± 23.60	56.70 ± 9.00
**TFN-2**	37.83±7.00	143.70±21.60	48.13±9.90
**TFN-3**	70.60 ± 13.50	227.50 ± 39.50	88.20 ± 16
**TFN-4**	80.60 ± 15.50	292.50 ± 45.30	99.60 ± 18.80

Ra (average roughness), Rz (mean height roughness) and Rrms (root mean square roughness).

Table S4. Parameters of inorganic ions in the feed solution [1,2].

Ions	Z	Hydrated radius (Å)	D (10^{-3} mm^2s^{-1})
Na^+	1	3.58	1.33
SO_4^2	-2	3.79	1.07
Mg^2	2	4.28	0.71
Cl^-	-1	3.32	2.03

References

1. H. Sun and P. Wu, "Tuning the functional groups of carbon quantum dots in thin film nanocomposite membranes for nanofiltration," *J. Membr. Sci.*, vol. 564, pp. 394–403, Oct. 2018, doi: 10.1016/j.memsci.2018.07.044.
2. D.-X. Wang, M. Su, Z.-Y. Yu, X.-L. Wang, M. Ando, and T. Shintani, "Separation performance of a nanofiltration membrane influenced by species and concentration of ions," *Desalination*, vol. 175, no. 2, pp. 219–225, May 2005, doi: 10.1016/j.desal.2004.10.009.

Disclaimer/Publisher's Note: The statements, opinions and data contained in all publications are solely those of the individual author(s) and contributor(s) and not of MDPI and/or the editor(s). MDPI and/or the editor(s) disclaim responsibility for any injury to people or property resulting from any ideas, methods, instructions or products referred to in the content.
